# Hypertension in Childhood Nephrotic Syndrome

**DOI:** 10.3389/fped.2019.00287

**Published:** 2019-07-16

**Authors:** Ibrahim F. Shatat, Lauren J. Becton, Robert P. Woroniecki

**Affiliations:** ^1^Pediatric Nephrology and Hypertension, Sidra Medicine, Doha, Qatar; ^2^Department of Pediatrics, Weill Cornell College of Medicine-Qatar, Doha, Qatar; ^3^College of Nursing, Medical University of South Carolina, Charleston, SC, United States; ^4^Private Practice Practitioner, Pediatric Nephrology, Seattle, WA, United States; ^5^Pediatric Nephrology and Hypertension, Stony Brook Children's Hospital, Stony Brook, NY, United States

**Keywords:** nephrotic syndrome, hypertension, pediatric, ambulatory blood pressure, blood pressure variability

## Abstract

Arterial hypertension (HTN) is commonly encountered by clinicians treating children with steroid sensitive (SSNS) and steroid resistant nephrotic syndrome (SRNS). Although the prevalence of HTN in SSNS is less documented than in SRNS, recent studies reported high prevalence in both. Studies have estimated the prevalence of HTN in different patient populations with NS to range from 8 to 59.1%. Ambulatory HTN, abnormalities in BP circadian rhythm, and measures of BP variability are prevalent in patients with NS. Multiple mechanisms and co-morbidities contribute to the pathophysiology of HTN in children with NS. Some contributing factors are known to cause acute and episodic elevations in blood pressure such as fluid shifts, sodium retention, and medication side effects (steroids, CNIs). Others are associated with chronic and more sustained HTN such as renal fibrosis, decreased GFR, and progression of chronic kidney disease. Children with NS are more likely to suffer from other cardiovascular disease risk factors, such as obesity, increased measures of arterial stiffness [increased carotid intima-media thickness (cIMT), endothelial dysfunction, increased pulse wave velocity (PWV)], impaired glucose metabolism, dyslipidemia, left ventricular hypertrophy (LVH), left ventricular dysfunction, and atherosclerosis. Those risk factors have been associated with premature death in adults. In this review on HTN in patients with NS, we will discuss the epidemiology and pathophysiology of hypertension in patients with NS, as well as management aspects of HTN in children with NS.

## Introduction

Nephrotic syndrome (NS) is one of the most common childhood kidney diseases worldwide, with a reported incidence of 2–16.9/100,000 children (1, 2). NS encompasses several primary and secondary renal diseases that have common physical changes in glomerular filtration barrier, which result in a massive leak of serum proteins into the urine. The great majority of cases are steroid responsive, with only <20% of children with NS being steroid resistant (3). Minimal change disease (MCD) is the most common glomerular pathology. Although MCD carries an excellent prognosis with low risk of progression to ESRD, its relapsing nature necessitates that children receive frequent courses of steroid therapy and other steroid-sparing medications, many of which are known to affect blood pressure (BP). NS was once thought to be associated with normal or low blood pressure, as described by Volhard's comment, “One of the most important criteria of nephrosis is absence of blood pressure elevation and absence of cardiac hypertrophy (4).” In fact, now we know that a significant portion of children with NS have HTN (Table 1).

**Table 1:** Summary of studies that reported on the prevalence of HTN in children with NS.

**Nephrotic syndrome**	**HTN prevalence/ other factors**	**Definition of HTN**	**Study (references) year of publication**
MCD (Nill disease) FSGS (focal glomerular obsolescence)	12.8% 13.4%	DBP > 98th %tile	ISKDC (6, 7) 1981
SRNS, congenital NS	10.2% in infants 27.9% in adolescents	ND	PodoNet (8) 2015
FSGS (SSNS) FSGS (SRNS)	14.3% 66.7%	ND	Shatat et al. (9) 2007
SSNS	23.4% in remission	SBP and/or DBP ≥ 95th %tile for gender, age, and height on ≥ 3 occasions	Keshri et al. (10) 2018
MCD	95% in relapse/edema 19% in remission	BP > 95th %tile for age	Küster et al. (11) 1990
SSNS	65% active phase 34% remission	BP > 90th %tile for age	Kontchou et al. (12) 2009
Glomerulonephritis/FSGS with GFR ≤ 75 ml/min/1.73 m^2^	17.5%	SBP or DBP ≥95th %tile for gender, age, and height	Mitsnefes et al. (13) 2003
FSGS with GFR > 40 ml/min per 1.73 m^2^	56.9% cyclosporine arm 59.1% MMF/Dex arm	History of HTN, or BP > 95th %tile for gender age, and height or >140/95 for adults	FSGS-CT (14) 2013
Nephrotic proteinuria Sub-nephrotic proteinuria	17% 60%	SBP ≥ 95th %tile for gender, age, and height	CKiDS (15) 2008
Nephrotic proteinuria Sub-nephrotic proteinuria	23% 54%	DBP ≥ 95th %tile for gender, age, and height	CKiDS (15) 2008
MCD FSGS	41% 46.9%	SBP or DBP ≥ 95th %tile for gender, age, and height	NEPTUNE (16) 2017
SSNS SDNS SRNS	7% 19.7% 12.6%	ND	Gabban et al. (17) 2010
SRNS	31.9%	Need for anti-HTN medication	Inaba et al. (18) 2016

*MCD, minimal change disease; FSGS, focal segmental glomerulosclerosis; NS, nephrotic syndrome; SSNS, steroid sensitive NS; SRNS, steroid resistant NS; SDNS, steroid dependent NS; SBP, systolic blood pressure; DBP, diastolic BP; %-tile, percentile; HTN, hypertension; ND, not defined*.

NS in children is classified by The American Heart Association as a Tier II (moderate) cardiovascular risk factor (5). The etiology of HTN in nephrotic syndrome (NS) is multifactorial; it is related to a host of both renal and non-renal intrinsic and extrinsic/environmental factors. Some contributing factors are known to cause acute and episodic elevations in blood pressure such as fluid shifts and medication side effects. Others are associated with chronic and more sustained HTN, including renal fibrosis, decreased GFR and progression of chronic kidney disease.

In this review, we examine the prevalence and pathophysiological factors that contribute to the development of HTN in children with NS. We focus our review on primary NS due to MCD and focal segmental glomerulosclerosis (FSGS). We discuss BP variability, circadian rhythms, and ambulatory blood pressure monitoring (ABPM) in this patient population, and highlight management considerations of HTN in children with NS.

## Epidemiology

The reported prevalence of HTN in childhood NS varies widely. Studies have used different HTN definitions, included patients at different states of NS disease activity, and recruited heterogeneous NS populations with varying glomerular filtration rates (GFRs) receiving different anti-proteinuric and antihypertensive medications, which has made it hard to discern an accurate prevalence of HTN in children with NS.

The International Study of Kidney Disease in Children (ISKDC) reported diastolic blood pressure > 98th %tile in 25/195 (12.8%) of children with Nil disease (MCD), and in 11/82 (13.4%) children with focal glomerular obsolescence (FSGS) (6, 7). Utilizing the PodoNet registry cohort, Trautmann et al. reported the prevalence of HTN at presentation in steroid-resistant and congenital NS to range from 10.2% in children <3 months to 27.9% in adolescents. Children with the histopathologic diagnosis of diffuse mesangial sclerosis had the highest prevalence of HTN (26.3%) (8). Previously, we have reported the prevalence of HTN in a small cohort of children with FSGS to be 43.8%. Steroid resistant patients had a much higher prevalence compared to steroid sensitive patients: 66.7 and 14.3%, respectively (9). Keshri et al. examined 81 children with SSNS in remission and off steroid therapy for 1–10 years and found 23% with hypertension. Of those, 73% had a positive family history of HTN compared to 32% in the normotensive group. BP was significantly correlated with serum cholesterol and LDL levels (10).

In a study examining the prevalence of hypertension in MCD and other types of NS, Küster et al. reported the pre-steroid treatment (edematous phase) prevalence of HTN (defined as BP > 95% of age) in 57 children to be 95%. After complete remission, the prevalence of HTN in this cohort decreased to 19% (11). Kontchou et al. examined the possible role of family history of HTN on the prevalence of HTN in children with MCD. Forty-nine prepubertal children with NS were included; 65% had systolic and/or diastolic BP > 90th %tile in the first week of edema. After 4 weeks of steroid therapy, 34% of children still had blood pressure > 90th percentile. They reported a much higher prevalence of HTN in children with MCD with family history of essential hypertension compared to children with no family history. This difference was more striking (88 vs. 53%) at disease presentation (edematous stage) but also persisted (52 vs. 34%) after 4 weeks of steroid therapy (12).

Mitsnefes et al. reported the prevalence of HTN (SBP or DBP ≥ 95th percentile for gender, age, and height) in a cohort of 3,834 children registered in NAPRTCS to be 48%. In a subgroup of 546 children with glomerulonephritis/FSGS, the prevalence was 17.5%. Children with HTN were more likely to reach the study endpoints (dialysis or 10-ml/min/1.73 m^2^ drop from baseline eGFR) compared to the normotensive children. It is important to point out that this cohort was composed of children with impaired kidney function and only 13.8% of the cohort had glomerulonephritis/FSGS (13). In children enrolled in the FSGS Clinical Trial, 41 out of 72 (56.9%) children randomized to cyclosporine, and 39 out of 66 (59.1%) children randomized to MMF/dexamethasone had HTN (14). Baseline blood pressures were not reported in this study. In children with nephrotic proteinuria enrolled in Chronic Kidney Disease in Children (CKiD) Study, 17% (10 subjects of 432) had systolic blood pressure ≥ 95th %tile and 23% (13/432) had diastolic blood pressure ≥ 95th %tile. HTN was more prevalent in children with sub-nephrotic proteinuria: 60% (35/432) for SBP and 54% (31/432) for DBP, respectively (15).

A recent review of 147 children enrolled in the Nephrotic Syndrome Study Network (NEPTUNE), consisting of 69 (46.9%) children with Minimal Change Disease (MCD), 49 (33.3%) with FSGS, 8 (5.4%) with IgA, 2 (0.04%) with Membranous Nephropathy, and 19 (12.9%) other glomerulopathy, showed that 69 (46.9%) had prior history of HTN or blood pressure ≥95th% at baseline visit. Authors reported no statistical difference in prevalence of hypertensive status between MCD (41%), FSGS (46.9%), and IgA (62.5%) subjects (16). This is possibly related to the preservation of kidney disease in the childhood years, lessening the effect of lower GFR usually seen with advancing age in FSGS patients. One other possibility is the fact that childhood MCD tends to have a relapsing course necessitating repeated courses of steroids and other steroid-sparing medications, which may increase prevalence of HTN over time.

Gabban et al. prospectively examined a cohort of 71 children 1–18 years old: 33 (46.5%) with steroid sensitive NS, 28 (39.4%) with steroid dependent NS, and 10 (14.1%) with steroid-resistant NS. HTN was present at diagnosis in 5 patients (7%) with steroid sensitive NS, 9 patients (12.6%) with steroid resistant NS and 14 patients (19.7%) with steroid dependent NS. Family history of HTN was reported in 4 cases (5.6%). In over 7 months of follow-up, the overall prevalence of HTN increased to 39.4% (17).

Inaba et al. followed 69 children 1–5 years of age with idiopathic steroid resistant NS for ≥4 years and found that HTN, defined as the need for anti-hypertensive therapy except when given for renoprotective purpose, affected 31.9% of patients (18).

## Ambulatory Hypertension, Abnormalities in BP Circadian Rhythm, Measures of BP Variability Are Prevalent in Patients With NS

Sarkar et al. examined in-clinic and ABPM in 99 children with frequently relapsing NS. Clinic blood pressure was >95th percentile in 63 (63.6%) patients. Ambulatory HTN was present in 33.3, 16.1% had masked HTN, and 30.3 % had white coat HTN. Nocturnal non-dipping was seen in 72 and 55 patients had a high nocturnal systolic BP load. Twenty-one patients had increased left ventricular mass index (LVMI), of which 42.9% had ambulatory HTN, 14.3% had masked HTN and 28.6% patients had white coat HTN (19). These circadian BP abnormalities and high prevalence of nighttime HTN were also reported in another smaller cohort of 21 patients with primary NS, 17 with MCD and 4 with FSGS. Of these patients, 8 (38%) had daytime HTN, 13 (62%) had nighttime HTN, and 13 (62%) were non-dippers. The data from repeated ABP measurements, before and after the achievement of remission, showed a marked decrease in the average 24 h ABP after remission (20). Xu et al. demonstrated by ABPM that among 114 children with primary NS, 101 had elevated BP (88.6%), 45 showed high incidence of masked HTN (39.5%), and 80 had (nocturnal) non-dipping BP (70.2%).

Data from the NEPTUNE study showed BP variability (BPV) to be associated with the study pre-determined endpoints (complete remission and composite endpoint of ESRD or loss of ≥40% GFR). Greater systolic and diastolic standard deviation (SD) and average real variability were associated with greater hazard of reaching the composite end point in adults (all *P* < 0.01). In children, greater BPV was an independent predictor of composite endpoint (determined by systolic SD and average real variability) and complete remission (determined by systolic and diastolic average real variability); all *P* < 0.05 (16).

## Comorbidities Contributing to the Development of Hypertension and Increased Cardiovascular Disease Risk in Children With NS

Children with NS suffer from comorbidities related to medication side effects and the NS disease pathophysiology, many of which are known to increase cardiovascular disease risk including obesity, left ventricular hypertrophy (LVH), increased measures of arterial stiffness (increased cIMT, endothelial dysfunction), impaired glucose metabolism, and hyperlipidemia.

Several studies have reported on the high prevalence of obesity and overweight in children with NS. In a 10 years follow-up study, Ishikura et al. reported 8% of children with frequently relapsing/steroid dependent NS to be obese (21). In a longterm follow-up study (from childhood to adulthood) of 61 patients with NS, Skrzypczyk et al. reported 23% of patients were overweight, 4.9% obese, and 16.1% were hypertensive as adults (22).

Hyperlipidemia, prevalent while children are having significant proteinuria, generally improves once NS is in remission. In a subset of NS patients with steroid resistance and/or frequent relapses, hyperlipidemia becomes a chronic condition. The disorders of lipid and lipoprotein metabolism in NS contribute to the development and progression of cardiovascular and kidney disease, and has been nicely reviewed elsewhere (23). Studies have shown the effects of lipid dysregulation to last for years after steroid treatment (24).

Patients with NS have increased arterial stiffness; Cungor et al. found significantly higher carotid-femoral pulse wave velocity (PWV) in adults with NS compared to healthy controls. Mean arterial pressure was predictive of arterial stiffness (25). Rahul et al. described a lower flow-mediated dilatation (FMD) in 32 children with NS disease duration of more than 2 years compared to their matched controls. The authors attributed the lower FMD to endothelial dysfunction (26). It is important to point out that the authors did not find significant differences in cIMT between the two groups. On the other hand, Hooman et al. found children with NS to have higher cIMT compared to healthy sex- and age-matched children. In their study, the duration of NS and systolic HTN were significantly correlated with carotid IMT (27).

Candan et al. demonstrated subclinical cardiovascular disease in 37 pediatric patients with steroid resistant NS. Compared with the controls, those patients had significantly higher mean aortic PWV, mean cIMT, and LVMI. Increased aortic PWV was noted in 5% of patients, increased carotid IMT in 22%, and increased LVM index in 19% while nocturnal dipping was absent in 67.6% (28).

In summary, the prevalence of HTN in children with NS is highly variable. This is largely due to the dynamic nature of NS disease process. Patients may be in relapses or remission, some patients require treatment with IV albumin infusion and diuretics, patents can be on different and changing doses of medications known to affect BP, and occasionally patients may develop acute kidney injury (AKI) as a complication of NS. Furthermore, studies did not use the same HTN definition, included patients at different states of the NS disease process, included heterogeneous NS populations with varying GFRs and receiving different anti-proteinuric and antihypertensive medications. Although studies examining arterial stiffness in this patient population have reported conflicting results, the majority have signaled an increased arterial stiffness (assessed by PWV, FMD, and cIMT) and highlighted the need for larger prospective controlled studies to further understand the prevalence and the pathophysiology of arterial stiffness in children with NS.

## Pathophysiology of Hypertension in Patients With NS

**Figure 1:**
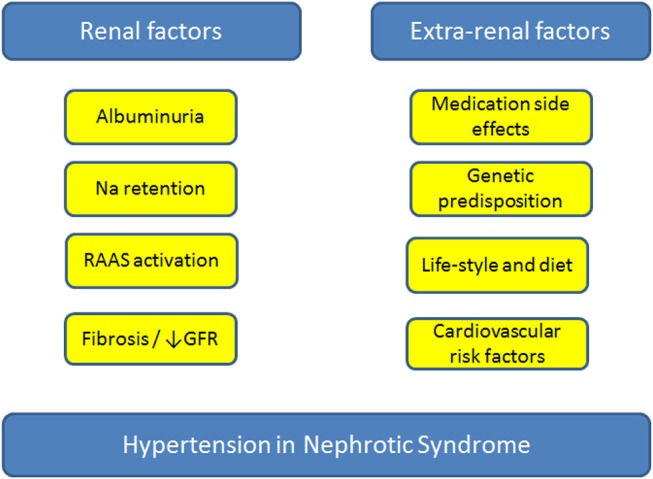
Factors contributing to the development of hypertension in patients with nephrotic syndrome. GFR, glomerular filtration rate; Na, sodium; RAAS, renin-angiotensin-aldosterone-system.

The pathophysiology of HTN in NS is complex, with multiple renal and extra-renal contributing factors. Renal factors include sodium retention, fibrosis/loss of GFR and progression of kidney disease, and recently, a feed-forward loop between albuminuria and blood pressure has been described (29). On the other hand, extra-renal factors include medication side effects, co-morbid conditions, and genetic predisposition (Figure 1).

Sodium handling in NS not only has a central role in the development of edema but also plays an important role in blood pressure regulation. Two main hypotheses have been posited to explain the development of sodium retention in NS: the underfill and the overfill hypotheses (30). In the underfill hypothesis, hypovolemia secondary to low oncotic pressure and third-spacing leads to activation of the renin-angiotensin-aldosterone system (RAAS) and sodium retention. In the overfill hypothesis, RAAS is suppressed and it is believed that the sodium retention is related to an intrinsic renal defect in sodium handling. Multiple investigators have examined the role of ENaC activation in sodium and water retention in NS. In those experiments, a defective glomerular filtration barrier allowed the passage of proteolytic enzymes that had the ability to activate ENaC (31, 32). ENaC mediates the absorption of sodium from the distal parts of the nephron. In NS, multiple factors contribute to the activation of these channels; aldosterone, vasopressin, proteases, and urinary plasminogen in tubular ultra-filtrate all appear to contribute to its activation. Activation of the ENaC not only enhances the development of edema, but also has implications on blood pressure regulation (30). To highlight the complexity, heterogeneity, and multifactorial nature of HTN in children with NS, Kuster et al. observed in 57 children with NS and despite administration of steroids, BP fell during treatment even before proteinuria had completely disappeared, suggestive of overfill (altered intrarenal sodium handling) at play (11).

In response to volume expansion, atrial natriuretic peptide (ANP) facilitates diuresis by increasing the GFR while reducing sodium reabsorption in the renal tubules. Investigators have demonstrated a blunted response to elevated serum ANP levels in NS (33–35). This blunted effect has been attributed to abnormal ANP-dependent signaling mechanisms and decreased conversion of pro-ANP to active ANP in proteinuric kidney disease (36).

Investigators have also examined the role of nitric oxide (NO) in sodium retention and the pathogenesis of HTN in animal models of NS. Ni et al. (37) demonstrated reduction in kidney NO synthase and reduced fractional excretion of sodium (FENa) in proteinuric rats. The authors attributed the NO synthase deficiency in their model to the presence of proteinuria, and thus the NO deficiency reduced FENa by augmenting renal tubular sodium reabsorption and preglomerular vasoconstriction.

Many of the commonly used medications in the treatment of NS are known to affect BP and contribute to the development of HTN. Calcineurin inhibitors induce nephrotoxicity and arterial HTN (38). Vasoconstriction, sympathetic excitation and sodium retention by the kidney had been shown to play a role in CNI induced HTN (38, 39). CNIs are usually used in SRNS patients; a subgroup of NS patients that are more likely to have HTN compared to the SDNS patients (9). In a retrospective study examining the effects of tacrolimus in treatment-resistant nephrotic syndrome, 6/15 children developed worsening or new-onset HTN (40). El-Husseini et al. reported the development of HTN in 10% of children with NS who received cyclosporine treatment for more than 2 years and 6% of children developed renal insufficiency (mostly patients with FSGS on renal biopsy) (41).

Synthetic steroids (prednisolone, prednisone, and methyl-prednisone) are central in the treatment regimens of patients with NS. These steroids have slightly different glucocorticoid and mineralocorticoid activities with predominantly glucocorticoid (immunosuppressive, anti-inflammatory properties); traditionally, it is their salt-retaining via mineralocorticoid-receptor properties that have been blamed for the effects on BP. The exact mechanism by which the glucocorticoid effect induces HTN is unclear. The high prevalence of HTN in steroid—treated patients has been reported even in therapy with predominantly glucocorticoid activity. Synthetic steroids may play a role in BP regulation via other mechanisms, such as fluid shifts from interstitial to the intravascular compartment, elevated plasma renin activity, increased sympathetic nerve activity, altered prostaglandin biosynthesis, enhanced vascular smooth muscle responsiveness to catecholamines and angiotensinogen II, impaired vasodilation, and nitric oxide synthase activity (42–47).

The effect of steroid therapy on BP in children with NS is variable. Some NS patients may develop HTN or worsening of HTN with steroid therapy, while others have improved BP after achieving remission despite being on high dose steroid therapy. Klepikov at al. described 7/27 NS patients who developed HTN with steroid therapy. They were more likely to be hypervolemic with severe sodium retention and suppressed renin and aldosterone levels. On the other hand, normo- and hypo-volemic patients had a robust diuresis and natriuresis with steroid therapy (48). Kontchou et al. reported improved BP profiles in children with MCD after 4 weeks of steroid therapy (12). The heterogeneity in the effects of steroids on BP maybe attributed to the heterogeneity of the study populations and the complex interplay between genetic and environmental factors.

Recently, a study by Haas at al. supported the existence of a feed-forward loop between albuminuria and blood pressure and implied that albuminuria could increase the risk of cardiovascular disease through blood pressure. In their study, the authors first performed a genome-wide association study for albuminuria in a large cohort and identified 32 new albuminuria loci. Then they constructed an albuminuria genetic risk scores and tested the score for association with cardiometabolic diseases. Genetically elevated albuminuria was strongly associated with increased risk of HTN. The authors also found that the relationship between BP and albuminuria was bidirectional; genetically elevated albuminuria led to higher BP and higher systolic BP predicted an increase in albuminuria. These findings suggest that pathways leading to albuminuria such as endothelial dysfunction, impaired kidney function and decreased ability to excrete sodium may contribute to the pathogenesis of HTN. Although the study was not conducted in a NS cohort, it does represent an additional significant step toward our understanding of the effects of albuminuria (the primary protein lost in patients with NS) on BP and highlights the need for further studies to better understand this complex interplay between albuminuria and HTN (29).

## Management of Hypertension in Patients With NS

Uncontrolled HTN is a well-known cardiovascular disease risk factor; in adults it is associated with cardiovascular morbidity and mortality (49, 50). In children, studies have shown HTN to be associated with target-organ damage (TOD), such as LVH, cognitive impairment, and faster progression of chronic kidney disease (15).

Salt restriction and RAAS inhibition are considered integral parts of the management of children with proteinuria and both are known to have a blood pressure reducing effect. RAAS blockade is known to have a reno-protective effect in patients with glomerular disease; some studies have demonstrated a greater reduction of proteinuria with combination ACEi/ARB therapy. This reno-protective effect has been attributed to both BP reduction and BP independent mechanisms (51, 52). Both the AIPRI and the REIN studies support that angiotensin-converting enzyme (ACE) inhibitors have a long-term renoprotective effect. The benefits of ACE inhibitors can be demonstrated even in patients who are not hypertensive (53, 54). It is important to point out that the use of ACEi/ARB therapy in an intravascularly depleted child may precipitate AKI. Treating clinicians should always weigh the risk and benefit before initiating therapy.

Studies examining the effects of blood pressure control in children with idiopathic NS are lacking. On the other hand, the benefit of strict BP control has been demonstrated in children with CKD. The ESCAPE trial (13.5% of study cohort had glomerulopathies) showed intensified blood-pressure control to confer a substantial benefit with respect to renal function (55).

Aldosterone is a known contributor to the sodium retention in patients with NS (56). Recently, effects that are independent of sodium transport have been described too, including increased fibrosis, collagen deposition, inflammation, and remodeling of the heart and blood vessels. These effects are markedly increased in the presence of high sodium intake (57). Studies have shown the addition of spironolactone (an aldosterone antagonist) to ACE inhibitor leads to further reduction of proteinuria, an effect that maybe confounded by BP reduction. In adult patients with CKD, spironolactone, when added ACE/ARB, was found to reduce proteinuria levels as well as the rate of GFR loss (58). Unfortunately, spironolactone treatment is associated with a significant increase in serum potassium levels, which necessities close electrolyte monitoring and limits its use in patients with lower GFRs.

Sparsentan, which combines endothelin receptor type A blockade with angiotensin II inhibition, has been shown to reduce proteinuria in patients with FSGS. It also had a greater effect on lowering BP compared with irbesartan (59).

Diuretics play an important role in the management of children with NS (60). Different classes of diuretics result in net fluid and sodium loss, a desired goal in the management of the edematous and hypertensive child. It is important to caution against aggressive diuresis of the intravascularly deplete child and the treating clinician needs to carefully weigh risks and benefits while using diuretics in combination with other BP-lowering medication classes such as RAAS inhibitors. As previously discussed, ENaC mediates the absorption of sodium from the distal parts of the nephron (30). ENaC inhibition with amiloride is known to reduce edema and improve blood pressure and has been reported to resolve edema and HTN in a patient with NS (61). However, the use of this agent may be limited by the risk of hyperkalemia.

In addition, while salt restriction and RAAS inhibition represent the key pillars in the management of the hypertensive child with proteinuria, other classes of antihypertensive medications have been used. While the use of beta blockers carries the theoretical benefit of lowering BP and indirectly inhibiting the RAAS without impairing the GFR, there are several reports suggesting worsened glycemic control with this class. Vasodilators, when used alone, may result in greater sodium retention. Unlike other vasodilating drugs, calcium channel blockers do not cause renal sodium and water retention, and do not cause hyperkalemia. One perceived advantage of CCBs may be their lack of nephrotoxicity, which makes this drug class an appealing choice for monotherapy or combination therapy in patients with kidney disease. However, the safety of some CCBs (dihydropyridine vs. non-DHP) in patients with proteinuric renal diseases (mostly adults patients with diabetic nephropathy) and renal insufficiency may be questioned because of reported untoward effects on urinary protein excretion (62, 63). Finally, it is important to emphasize the importance of lifestyle interventions (exercise and dietary counseling) as part of HTN management in this patient population.

Given the heterogeneity in intravascular volume status of patients with NS (volume expansion and low renin in a subset of patients while others have intravascular depletion and elevated renin levels), the management of the hypertensive child with NS should aim at addressing possible contributing factors, taking into consideration the child's clinical examination, laboratory findings and current medications.

## Summary

HTN is prevalent in childhood NS. The increased risk of HTN lasts many years after stopping therapy and remission of the disease. Clinicians should continue to monitor blood pressure in children with a history of NS. Multiple factors are known to affect BP and contribute to the development of HTN in NS. Treatment of HTN should address the underlying disease pathophysiology and include lifestyle modifications. HTN and the cardiovascular disease risk in children with NS may be under recognized. 24-h ABPM can identify abnormalities in blood pressure measurements and patterns in this at-risk population. The majority of existing studies that examined the prevalence of HTN in patients with NS were observational and included heterogeneous NS patient populations, which makes it challenging to draw clear conclusions about the true prevalence and explains the substantial variability in the reported prevalence (Table 1). HTN in children with NS remains poorly understood and there is a pressing need for studies in this patient population to identify the “optimal” safe and efficacious antihypertensive medication class.

## Future Directions

Children with HTN and NS are at increased risk for dyslipidemia, LVH, left ventricular dysfunction, atherosclerosis, and aortic stiffness, all factors that have been associated with premature death in adults (64). Future research should aim at exploring specific patient characteristics that predispose a subset of children with NS to develop HTN; this should be carried out using well-defined patient populations and using a standardized BP definition. Children may benefit from already-proven measures to reduce cardiovascular disease risk in adults: diet, exercise, avoidance of smoking/vaping/illicit drugs, stress reduction, lipid lowering agents, cardiac remodeling agents, and renoprotective drugs (65). Another area in need of further research is the role of ABPM in the assessment of BP in children with NS. The current clinical practice guidelines for screening and management of HTN in children do not address ABPM indications in children with SSNS or SRNS with normal GFR (66). The role of immune pathways and their role in the pathogenesis of HTN in NS, a disease that is widely believed to have underlying immune dysregulation, warrant more investigation. Recently, the human microbiome role in BP regulation has been described (67). Future research is needed to better understand the relationship between the human microbiome and virome in BP regulation in children with NS. Currently, a multi-centric prospective cohort study of 70 steroid-resistant, 70 steroid-sensitive, and 70 healthy controls are being recruited to better understand the epidemiology of endothelial dysfunction and associated subclinical cardiovascular co-morbidity in childhood NS (68).

## Author Contributions

All authors listed have made a substantial, direct and intellectual contribution to the work, and approved it for publication.

### Conflict of Interest Statement

The authors declare that the research was conducted in the absence of any commercial or financial relationships that could be construed as a potential conflict of interest.
